# Molecular, behavioural and morphological comparisons of sperm adaptations in a fish with alternative reproductive tactics

**DOI:** 10.1111/eva.13438

**Published:** 2022-07-02

**Authors:** Charlotta Kvarnemo, Leon Green, Ola Svensson, Kai Lindström, Sofie Schöld, Martina Griful‐Dones, Jonathan N. Havenhand, Erica H. Leder

**Affiliations:** ^1^ Department of Biology and Environmental Sciences University of Gothenburg Gothenburg Sweden; ^2^ Centre for Marine Evolutionary Biology University of Gothenburg Gothenburg Sweden; ^3^ Department of Educational Work University of Borås Borås Sweden; ^4^ Environmental and Marine Biology Åbo Akademi University Turku Finland; ^5^ Swedish Meteorological and Hydrological Institute Norrköping Sweden; ^6^ Department of Biology University of Barcelona Barcelona Spain; ^7^ Department of Marine Sciences University of Gothenburg Gothenburg Sweden; ^8^ Department of Biology University of Turku Turku Finland; ^9^ Natural History Museum University of Oslo Oslo Norway

**Keywords:** accessory glands, gene expression, Gobiidae, mucins, sexual selection, sperm competition, sperm performance, spermatozoa

## Abstract

In species with alternative reproductive tactics, there is much empirical support that parasitically spawning males have larger testes and greater sperm numbers as an evolved response to a higher degree of sperm competition, but support for higher sperm performance (motility, longevity and speed) by such males is inconsistent. We used the sand goby (*Pomatoschistus minutus*) to test whether sperm performance differed between breeding‐coloured males (small testes, large mucus‐filled sperm‐duct glands; build nests lined with sperm‐containing mucus, provide care) and parasitic sneaker‐morph males (no breeding colouration, large testes, rudimentary sperm‐duct glands; no nest, no care). We compared motility (per cent motile sperm), velocity, longevity of sperm, gene expression of testes and sperm morphometrics between the two morphs. We also tested if sperm‐duct gland contents affected sperm performance. We found a clear difference in gene expression of testes between the male morphs with 109 transcripts differentially expressed between the morphs. Notably, several mucin genes were upregulated in breeding‐coloured males and two ATP‐related genes were upregulated in sneaker‐morph males. There was a partial evidence of higher sperm velocity in sneaker‐morph males, but no difference in sperm motility. Presence of sperm‐duct gland contents significantly increased sperm velocity, and nonsignificantly tended to increase sperm motility, but equally so for the two morphs. The sand goby has remarkably long‐lived sperm, with only small or no decline in motility and velocity over time (5 min vs. 22 h), but again, this was equally true for both morphs. Sperm length (head, flagella, total and flagella‐to‐head ratio) did not differ between morphs and did not correlate with sperm velocity for either morph. Thus, other than a clear difference in testes gene expression, we found only modest differences between the two male morphs, confirming previous findings that increased sperm performance as an adaptation to sperm competition is not a primary target of evolution.

## 1 INTRODUCTION

Sperm competition occurs when sperm from two or more males are competing to fertilise the same set of eggs (Parker, 1970, 1998). Based on sperm competition theory (Parker, 1990a, 1990b), males of species that reproduce under high risk of sperm competition are expected to invest more into ejaculate expenditure (e.g. larger testes, allowing ejaculates with greater numbers of sperm) than males of species that are exposed to sperm competition less often. There is abundant empirical support among species for males evolving larger testes, ejaculates with greater numbers of sperm, larger, faster or more consistent sperm in response to greater risk of sperm competition (Fitzpatrick et al., 2009; Lüpold et al., 2020; Stockley et al., 1997).

Comparisons can also be made *within* species, for example, between males adopting alternative reproductive tactics, such as dominant males that attract females and to a great extent are able to keep parasitically spawning males at bay, versus subdominant males that do not invest in attracting females and instead resort to sneaking or other forms of parasitic reproduction. The latter type of male faces a very high risk of reproducing under sperm competition (probability close to 1) and is, therefore, adapted to invest more in current ejaculations, for example, by having larger testes that produce more sperm (Parker, 1990b, 1998). Sneaker males may also be expected to show traits such as longer living or faster swimming sperm, to compensate for disadvantages in terms of female monopolisation, position or timing of ejaculation (Ball & Parker, 1996; Parker, 1998; Snook, 2005; Taborsky, 1998), although the theoretical basis for these predictions is considerably weaker than that for sperm numbers (reviewed by Kustra & Alonzo, 2020). There is strong empirical support for sneaker males having larger testes and producing ejaculates with higher sperm densities or numbers than dominant males (e.g. Kustra & Alonzo, 2020; Montgomerie & Fitzpatrick, 2009). In contrast, the support for the expectation that sneaker males would have sperm that outperform those of dominant males (in terms of sperm longevity, motility or velocity) is more mixed (Kustra & Alonzo, 2020). Still, the number of species included in Kustra and Alonzo's recent review is less than 30. Thus, adding more species to that list is important for a better understanding of performance‐related adaptations of sperm for males of alternative reproductive tactics.

Since the typical sneaker male does not defend a territory or provide parental care, it may be able to mate more often than dominant males. Hence, their evolution of large testes may also reflect a potentially greater utility of a sustained sperm supply (Parker & Pizzari, 2010). In contrast, dominant males invest resources in display traits, territorial defence and sometimes parental care, and a trade‐off between pre‐ and postcopulatory investment can be expected to result in dominant males having smaller ejaculates, slower swimming sperm, lower sperm viability or percentage of motile sperm (Kvarnemo & Simmons, 2013; Parker et al., 2012). Trade‐offs also exist among sperm traits, such as between sperm performance and longevity (Levitan, 2000; Snook, 2005; Yamamoto et al., 2017). For example, in the shell‐brooding cichlid *Lamprologus callipterus*, bourgeois males are too large to fit inside the shells where females spawn, yet parasitically spawning dwarf males fit inside and thus are able to release their sperm closer to the female than the bourgeois males. Accordingly, dwarf males produce sperm that swim straighter, but are significantly shorter‐lived, compared with sperm of bourgeois males (Taborsky et al., 2018).

Sperm velocity is expected to correlate with sperm morphology. Although longer sperm do not necessarily swim faster, the tail‐to‐head length ratio can relate to swimming speed (Humphries et al., 2008), which may differ between males with different reproductive tactics (Taborsky et al., 2018).

Resources invested in ejaculates do not only concern sperm numbers or sperm traits. Seminal fluid and other nongametic components of ejaculates that are produced by accessory glands can also markedly affect reproductive success (Cameron et al., 2007; Poiani, 2006). Such components can have multiple functions. Among other things, they are known to activate and energise sperm, affect female oviposition rate and receptivity, and influence microbial activity around sperm and eggs (e.g. Chapman, 2001; Chapman et al., 1995; Fitzpatrick & Lüpold, 2014; Giacomello et al., 2008; Poiani, 2006).

Sperm of many externally fertilising fishes have a very short lifespan after activation, on the timescale of seconds to minutes (Cosson, 2004; Dzyuba & Cosson, 2014). However, in the Family Gobiidae, multiple species show sperm lifespans that exceed hours, and even days (Green & Kvarnemo, 2019; Lindström et al., 2021; Locatello et al., 2007; Nakanishi & Takegaki, 2019; Scaggiante et al., 1999). In the same Family, males also have accessory glands (referred to as sperm‐duct glands) that produce a protein‐rich, mucus‐based substance that has an important impact on the micro‐environment around the gametes (Fishelson, 1991; Young & Fox, 1937). Typically, male gobies build enclosed nests using a substrate, such as a stone or a shell, in which they prepare an area for females to deposit their eggs by laying trails of mucus that contain pockets of embedded sperm (Marconato et al., 1996; Ota et al., 1996; Svensson & Kvarnemo, 2005). These embedded sperm are then gradually activated and released as the mucus dissolves (Marconato et al., 1996). Female gobies lay numerous eggs and attach them one‐by‐one to the nest substrate, which can take hours (Scaggiante et al., 1999). When multiple females spawn sequentially in the same nest, this egg‐laying phase becomes even longer.

Gradual sperm release from the mucus, together with long‐lived sperm, releases the nest‐holding male from having to stay very close to the female during the long period of egg‐laying, allowing the male to better protect the nest‐opening against sneaker males and egg predators (Marconato et al., 1996; Scaggiante et al., 1999). However, sneaker males may also have long‐lived sperm. Accordingly, in *Bathygobius fuscus*, nest‐holding males have been shown to remove sneaker male sperm from the nest by vigorously fanning the tail (Takegaki et al., 2020).

The sand goby, *Pomatoschistus minutus* (Gobiidae, Teleostei; Pallas 1770), is a valuable model for studies of reproduction in evolutionary ecology, not least because of well‐studied adaptations related to paternal care (e.g. Björk & Kvarnemo, 2011; Olsson et al., 2016; Pampoulie et al., 2004) and female choice (e.g. Forsgren, 1997; Lindström et al., 2006). Sperm competition is a ubiquitous part of its mating system, with parasitically fertilised eggs found in every second field‐collected nest studied, in which on average 22% of the eggs were fertilised by males other than the nest‐holding male (Jones, Walker, Kvarnemo, et al., 2001a; Jones, Walker, Lindström, et al., 2001b). Sand goby lifespan is unknown, but they appear to only have one breeding season, during which they spawn repeatedly, but grow very little (Kullander et al., 2012; Kvarnemo et al., 2010). In laboratory studies, males of all sizes, even egg‐guarding males, may spawn parasitically and fertilise eggs (Järvi‐Laturi et al., 2011; Singer et al., 2006; Svensson & Kvarnemo, 2007). However, some parasitically spawning males belong to a distinct sneaker‐morph, characterized by small body size, a lack of breeding colour, huge testes and rudimentary sperm‐duct glands (Kvarnemo et al., 2010; Svensson & Kvarnemo, 2007). It is unclear whether the sneaker‐morph is fixed for life, as it is for dwarf males in *L. callipterus* (Wirtz Ocana et al., 2014). In the sand goby, breeding morphology shows some plasticity depending on social environment, with sneaker‐morph males being able to build a nest, spawn with a female, and provide parental care, after prolonged experimental isolation from larger males (Malavasi et al., 2001; Takegaki et al., 2012). However, both behaviourally and morphologically, the transition is slow and incomplete, so the sneaker‐morph is likely to be genetically influenced or environmentally determined at an early age. In contrast to sneaker‐morph males, most breeding‐coloured males readily build a nest (a cave formed under, for example, a mussel shell or a stone, which they cover by sand), to which they sequentially attract several females to spawn by fin displays and courtship sounds (reviewed in Blom et al., 2016; Forsgren, 1999; Lindström & Lugli, 2000). Successfully mated males care for the eggs until hatching, by guarding, fanning and cleaning the eggs (Lindström & Wennström, 1994; Lissåker & Kvarnemo, 2006). Breeding‐coloured males vary in size, but are on average larger and have significantly smaller testes and larger sperm‐duct glands than sneaker‐morph males, irrespective of whether they hold a nest site (Kvarnemo et al., 2010; Svensson & Kvarnemo, 2007). Nest‐holding male sand gobies prepare the spawning area with sperm‐containing mucus trails, and in the visual presence of a sneaker male, nest‐holding males increase the rate of mucus trail deposition (Svensson & Kvarnemo, 2005). Adding the mucous contents of the sperm‐duct glands to the seawater has been shown experimentally to enhance sperm velocity of breeding‐coloured males (Green & Kvarnemo, 2019), but its effect on the sperm of sneaker‐morph males is unknown.

Marine model organisms are few and far between. To address this, the Centre for Marine Evolutionary Biology (CeMEB) included the sand goby among eight species for whole genome sequencing, with the aim to build marine model systems with a broad representation across the tree of life (www.gu.se/en/cemeb‐marine‐evolutionary‐biology/imago; Leder et al., 2021). The present study builds on this CeMEB initiative, focusing on the mechanisms of within‐species variation in reproductive tactics, comparing sperm traits and gene expression of breeding‐coloured males and sneaker‐morph males of the sand goby. Specifically, we compared sperm from breeding‐coloured males and sneaker‐morph males and examined if they differed in sperm motility (i.e. proportion motile sperm) and sperm velocity (swimming speed along a smoothed curvilinear path) at two different times (5 min and 22 h after activation). We used motility after 22 h to provide measures of sperm longevity. Twenty‐two hours was chosen based on a previous study that found sperm to be motile for ≥22 h in the sand goby and two congeners (Lindström et al., 2021). In a second experiment, we measured the same sperm performance traits in the presence or absence of the fluid from the sperm‐duct gland. We also compared the morphology of sperm and testes gene expression between breeding‐coloured and sneaker‐morph males.

Behavioural variation is an obvious trait related to alternative reproductive tactics. Consequently, most molecular research on alternative reproductive tactics has focused on brain mRNA expression and hormone levels (e.g. Aubin‐Horth et al., 2005; Renn et al., 2008; Somerville et al., 2019; Stiver et al., 2015). There is one study comparing gene expression in testes in a system analogous to our breeding‐coloured and sneaker males. It identified morph‐specific transcriptome signatures of both testes and brains in bluehead wrasses, with 40 genes being differentially expressed between sneaker and territorial males in the testes (Todd et al., 2018). Since we are interested in sperm‐trait‐mediated mechanisms by which sneaker males can compete with breeding‐coloured males, we examined mRNA expression in the testes using 3' RNA sequencing in addition to directly measuring sperm traits.

Based on the findings of previous studies (summarized in Kustra & Alonzo, 2020, and above) we made the following predictions. First, we expect sperm of sneaker‐morph males to exhibit traits associated with improved competitive ability, that is, higher longevity, motility and/or velocity, and greater flagella length to head length ratio, compared with sperm of breeding‐coloured males. Second, because only breeding‐coloured males have sperm‐duct glands and produce mucus‐based nest‐lining, we expected that sperm from these males would benefit from the presence of fluid from the sperm‐duct gland while sneaker‐morph sperm would show no difference or even be hampered by this fluid (but see Locatello et al., 2013). Finally, comparing the gene expression of testes of breeding‐coloured and sneaker‐morph males, we predicted genes associated with improved competitive ability in the form of increased sperm velocity, longevity or overall sperm production would be upregulated in sneaker‐morph males compared with breeding‐coloured males.

## 2 MATERIAL AND METHODS

### 2.1 Site of study, animal husbandry and dissections

The study was done at the Kristineberg Marine Research Station, on the Swedish west coast during two separate experimental runs. Sand goby males were caught from Bökevik, Fiskebäckskil, Sweden (58°14′54.1″N 11°26′48.0″E), a nearby natural breeding area, using a hand trawl. For the first experiment, fish were collected 23 May 2012, dissected 25–26 May and sperm variables recorded 25–27 May. For the second experiment, fish were caught 3, 6 and 7 May 2021, dissected 10–11 May and sperm variables recorded 10–12 May.

During both experiments, fish were kept in storage tanks (approx. 50 L with ≤20 males in each) for 2–7 days, before sampling. Although the males were isolated in single‐sex aquaria during this time, they had visual access to females in nearby aquaria. All storage tanks were supplied with running seawater pumped directly from the sea at 5 m depth, with a mean (± SE) temperature of 11.8 ± 1.4°C (2012) and 10.7 ± 0.3°C (2021) and a fluctuating salinity of 26.6 ± 2.8 PSU (2012) and 26.4 ± 0.5 PSU (2021). Each tank had a 3 cm layer of sand for the gobies to burrow in. The fish were fed a mix of frozen chopped mussel meat, brown shrimp, cod or Alaska pollock daily, and all tanks were cleaned before feeding.

### 2.2 The two experiments

The first experiment (2012) aimed to test the differences in sperm motility and velocity between breeding‐coloured and sneaker‐morph males over time, and to investigate how gene expression in testes differs between the two morphs. One testis from each male was used for analysis of sperm motility and sperm velocity. The other testis was snap‐frozen in liquid nitrogen for gene expression analysis. The second experiment (2021) aimed to investigate the influence of the contents of the sperm‐duct glands on sperm motility and velocity over time in the two male morphs. In 2021, we also measured sperm morphometrics of the two morphs.

#### 2.2.1 Sperm motility and velocity (2012 and 2021)

In both years, euthanasia was performed by a blow to the head, followed by decapitation and destruction of the brain using a scalpel. After this, the male was immediately dissected, and testes and sperm‐duct glands were removed and separated within 1 min, with the aid of a dissection microscope (6× magnification, M3 Wild Heerbrugg, Gais, Switzerland), stainless steel forceps and scissors.

In 2012, 10 breeding‐coloured males and 10 sneaker‐morph males were sampled in random order from the holding tanks. In 2021, the sampling order alternated between the two morphs, but within each morph males were selected randomly. For each morph, we labelled the males 1–10 according to the order they were sampled. To record sperm motility and velocity in the first experiment (2012), one testis of each male was placed into a 0.5‐ml microcentrifuge tube (Eppendorf, Hamburg, Germany), ruptured with a scalpel and 20 μl calcium‐free Ringer's solution (Karila et al., 1993) was added to prevent activation. From this fluid, 8 μl was removed and a stock suspension of activated sperm was created by re‐suspending this into a tube with 750 μl of filtered natural seawater (31.8 PSU: one tube for each male; kept in a rack surrounded by water of 12.5 ± 0.5°C for the whole study). The sperm motility assay followed that of Havenhand and Schlegel (2009) and developed in Green and Kvarnemo (2019). To ensure an accurate estimate of sperm movement from each male, time and treatment, we used average values of 3 technical replicates that were placed on the same microscope slide. Each technical replicate was created by placing 40 μl of sperm stock solution on an albumin‐coated microscope slide fitted with a 0.75‐mm‐thick O‐ring and capped with a coverslip. Sperm movement was recorded within <5 min from dissection, for 0.5 s at the midpoint of the drop, at 30 frames s^−1^, using a digital video camera (Pixelink, model PL‐D725, Ottawa, Ontario, Canada) mounted on an inverted microscope (Leica DM‐IL). Pilot experiments showed illumination by the microscope lamp had no impact on the temperature in the sperm suspension on the slide during video recording (time for illumination of slide was approx. 10 s). To understand the effect of time on sperm movement, each stock solution was resampled with another three technical replicates after 22 ± 1.5 h. For eight males (three sneaker‐morph males and five breeding‐coloured males) that still had sufficient stock sperm suspension remaining, a third sampling was performed at approx. 48 h after dissection, taking 2–4 technical replicates per stock solution.

The second experiment (2021) used a similar methodology. Testes of a breeding‐coloured male were dissected and placed into two separate 0.5 ml microcentrifuge tubes, one testis with, and one without, a sperm‐duct gland from the same male, creating two different treatments: a control and a sperm‐duct gland treatment. The second sperm‐duct gland was placed in a separate 0.5‐Eppendorf tube with 5 μl of Ringer's solution to keep it moist and held at 12°C, while the testes of a sneaker‐morph male were dissected to test the effect of sperm‐duct gland contents on the sperm of sneaker‐morph males. Thus, one testis from the sneaker‐morph male was placed with, and one without, the sperm‐duct gland from the breeding‐coloured male. The testis and gland (when present) were incised five times each using scissors, and the contents were diluted with 60 μl Ringer's solution at 12°C. From each of these sperm suspensions, 45 μl was taken and diluted into 750 μl of filtered seawater, creating a stock solution of activated sperm. Three drops, 40 μl each, of the stock solution were placed on a microscope slide and immediately filmed for three consecutive technical replicates. Sperm movement was recorded <5 min from dissection, for 0.5 s at the midpoint of the drop, at 30 frames s^−1^, using a Pixelink digital video camera, but here mounted on a different microscope (AxioVert.A1, Carl Zeiss AG, Oberkochen Germany). These two treatments were subsequently diluted with Ringer's solution and incised for sperm release as above, and the suspension activated in filtered seawater and filmed following the same protocol. After filming, 45 μl was taken from the stock solution of the control treatment and put into a 150 μl 4% formaldehyde solution for later sperm morphometrics measurements. A corresponding 45 μl was also removed from the stock solution of the sperm‐duct gland treatment to equalize volumes between the treatments that were kept for the second filming. The sperm stock solutions were held in their Eppendorf tubes at 12°C overnight, and after 22 ± 1.5 h, the sperm were filmed again using the protocol described above.

In both experiments, videos were postprocessed and analysed with ImageJ (National Institutes of Health) and using the CASA plugin (Wilson‐Leedy & Ingermann, 2007) to determine parameters related to sperm movement. Only sperm moving faster than 15 μm s^−1^ were included in the analysis, and replicates were inspected for circular paths indicative of functional disturbance limiting sperm progression (none were found). The person carrying out the CASA in 2012 was uninformed of (hence blind to) any expected outcome, and in 2021, the person was blind to which male morph, time and treatment each sample belonged to. In addition, since CASA is an automated procedure, which objectively measures sperm movement according to analysis parameters that were identical for all treatments and experiments, the risk of unconscious bias according to expectations is very low. Of all the output traits from CASA, per cent motile sperm and velocity (mean sperm velocity along a smoothed curvilinear path, VCL) were chosen for further analysis, since these traits have been found to correlate with fertilisation in several teleosts of different clades (Casselman et al., 2006; Gage et al., 2004; Gasparini et al., 2010; Stockley et al., 1997), including gobies reproducing in saltwater (Green, Niemax, et al., 2021b; Locatello et al., 2013). These traits have also been researched in several other studies of both sand gobies (Green & Kvarnemo, 2019; Lindström et al., 2021; Svensson et al., 2017) and other gobies (e.g. Green et al., 2019, 2020; Green, Apostolou, et al., 2021a; Lindström et al., 2021; Poli et al., 2018), making them suitable for within and cross‐species comparisons. All our results were qualitatively identical when using straight line velocity (VSL) as response variable instead.

#### 2.2.2 Sperm morphometrics (2021)

In 2021, sperm morphometrics were determined using a microscope (Leitz DM RBE, Leica Camera AG, Wetzler, Germany) fitted with a 100× objective (PL FLOUTAR 100×/1.3 oil PH3, Leica Camera AG, Wetzler, Germany) and a digital camera (Axiocam 705 colour, Carl Zeiss AG, Oberkochen Germany) illuminated with an external 100 w light bulb. Samples were prepared by placing 15 μl of suspended sperm solution on a microscope glass slide and adding a cover slip. An oil drop was then added to allow focusing at the selected magnification. The camera was manually focused on the surface of the glass slide, and the first 20 sperm with their tails perpendicular to the optic path were photographed. Morphometrics (head length including midpiece, and flagella length) were measured in the software ZEN 3.1 (Carl Zeiss AG, Oberkochen Germany) with a calibrated scale slide photographed at the same settings. The person carrying out these measurements was blind to male morph.

#### 2.2.3 Gene expression (2012)

RNA was extracted from the tissue of one testis each of the breeding‐coloured and sneaker‐morph males that were analysed in 2012, with a phenol‐based, phase separation method (Tri‐reagent, Ambion, Life Technologies) following the manufacturer's protocol and using 1‐bromo‐3‐chloropropane (SIGMA) instead of chloroform. RNA pellets were washed twice with 75% EtOH before resuspension. After resuspension in 30 μl nuclease‐free water, DNAse treatment with RQ1 (RNAse‐free DNAse, Promega) was performed to ensure no traces of DNA were left in the RNA. To further purify the RNA, samples were extracted for a second time with amounts of reagents downscaled for 400 μl of Tri‐reagent. The RNA samples were dissolved into 30 μl of nuclease‐free water. The RNA concentration was measured with Nanodrop prior to analysing quality on the Bioanalyzer (Agilent). RNA was prepared for sequencing using the QuantSeq 3' mRNA‐Seq Library Prep Kit FWD from Lexogen. Sequencing was performed on the Illumina HiSeq2500 at the Finnish Microarray and Sequencing Centre in Turku, Finland, using 50 base, single‐end sequencing.

Sequences were processed with cutadapt v2.7 (Martin, 2011) to remove partial adapters and bad‐quality sequences. Reads shorter than 40 bases were removed. The sand goby genome and transcript junctions for testes from Leder et al. (2021) were used as the genome for the STAR aligner (Dobin et al., 2013). STAR was run in quant mode to obtain gene counts. Read counts were imported into R (v4.0.3). EdgeR (v3.32.1; Robinson et al., 2010) was used to normalize expression using the TMM method (Robinson & Oshlack, 2010) and remove genes with low expression (minimum count = 10, minimum total count = 200). Then, a linear model, limma voom analysis (Law et al., 2014), was implemented using the R package limma (v3.46.0; Ritchie et al., 2015) to assess differential expression. Transcripts were considered differentially expressed if the adjusted *p*‐value was less than 0.05 using the Benjamini–Hochberg procedure (Benjamini & Hochberg, 1995). Fasta sequences were extracted for the significant transcripts using Gffread (Pertea & Pertea, 2020) from the testes gtf file (Leder et al., 2021), and BLASTX (2.11.0+ NCBI) was performed to obtain gene information. Functional enrichment was performed using gProfiler (Raudvere et al., 2019), using human identifiers (HGNC) through gene name searches in uniProt or BLASTX of the transcript but restricting hits to human genes.

### 2.3 Statistical analysis

For the 2012 data, we used chi‐square tests to analyse frequency data related to sperm longevity at 5 min, 22 h and 48 h. For the 2021 data, we instead estimated sperm longevity as the point in time when the sperm motility of a male would drop to 5% of the initial percentage of the motility measured at 5 min. This value was calculated for each male by linear equation based on the motility values at 5 min and 22 h and compared between male morphs using a one‐factor analysis of variance (ANOVA, linear model, *lm*).

Sperm motility and sperm velocity measurements from the three technical replicates were averaged to obtain mean values for each male (within a particular treatment and time), which were then treated as biological replicates in the statistical analysis. All statistical modelling of sperm performance was conducted in R (version 3.6.2) using the packages *lme4* (Bates et al., 2015), *lmerTest* (Kuznetsova et al., 2017) and *car* (Fox et al., 2013). Because the two experiments differed slightly in design (Table 1 for overview), the data from the years 2012 and 2021 were analysed separately. For the 2012 data, predictor variables modelled as fixed effects were male morph (breeding‐coloured or sneaker‐morph) and time (5 min or 22 h). For 2021, sperm‐duct gland treatment (with or without sperm‐duct gland contents) was added to the model as a third fixed factor. Models were analysed in a full factorial design with all interactions available. The linear versions of the models were visually explored by inspecting the residuals versus fitted values, theoretical and observed quantiles, high influence points and the frequency distribution of residuals, all using the ‘*plot(model)*’ function. The models were also assessed for variance inflation factors using the ‘*vif(model)*’ function, and none of concern was found. Both response variables (sperm motility and sperm velocity) were normally distributed and met assumptions of sphericity. Since sperm from each male were tested in multiple treatments and times, male identity was included as a random factor. The influence of the random factor was tested by a comparison between the linear and mixed models using the ‘*anova(linearmodel*, *mixedmodel)*’ function. For both response variables, the differences between models with and without the random term were significant. Mixed models were also consistently found to have better fit (judged by differences in AIC) than the linear models. The random factor was, therefore, kept in the final models. Where *p*‐values were of interest, *lmer* models were fitted with an estimated *p*‐value using Satterthwaite approximation in the R package *lmerTest*.

**TABLE 1:** Overview of results regarding motility (per cent motile sperm) and velocity (velocity of the curvilinear path, VCL) of sand goby (*Pomatoschistus minutus*) sperm measured in 2012 and 2021^a^

	2012		2021		
	Male morph	Time	Male morph	Sperm‐duct gland contents	Time
Sperm motility	No effect (0.74)	No effect (0.31)	No effect (0.88)	Nonsignificant trend towards higher when present (0.096)	Decline with time (<0.001)
Sperm velocity	No effect (0.53)	Nonsignificant trend towards decline with time (0.077)	Higher for sneaker‐morph males (0.031)	Higher when present (0.007)	Decline with time (<0.001)

^a^ In 2012, effects of male morph (sneaker male or breeding colour) and time (5 min and 22 h, as repeated measure) on motility and velocity were tested in seawater, in the absence of sperm‐duct gland contents. In 2021, the effect of sperm‐duct gland contents (absent or present, in the seawater) was tested, in addition to male morph and time (same as for 2012). *N* = 10 for each treatment. *p*‐Values are given in brackets.

We performed a principal component analysis on the 109 genes that differed in expression for breeding‐coloured and sneaker‐morph males. The expression value of each gene was first standardized to the mean using the *scale* function in R before it was entered into the analysis. We then analysed the extent to which gene expression captured by the first principal component (PC1) explained the observed variation in motility and velocity among males. For this, we used an analysis of covariance (ANCOVA) with PC1 as covariate, male morph as fixed factor, and (tested separately) sperm motility or velocity as dependent variable. This approach allowed us to evaluate whether the relationship between gene expression and sperm motility or velocity differed between male morphs, expressed as a significant interaction between gene expression and male morph.

Sperm total length was calculated by adding together the head and flagella length for each sperm. Average values for each individual male were then calculated, and all four measurements (head length, flagella length, total length and flagella length to head length ratio) were analysed using a Welch two‐sample *t*‐test using the ‘*t.test*’ function in R. The potential influence of sperm morphometrics on sperm velocity was tested using ANCOVA, with sperm velocity as dependent variable, male morph as factor, and (tested separately) sperm head length, flagella length, total length or flagella length to head length ratio as covariate. For each male, we used the value of sperm velocity that was measured in the control treatment, after 5 min.

### 2.4 Data archiving statement

We comply with Evolutionary Applications' policy on data archiving. Raw data supporting this study are available at the Dryad Data repository https://doi.org/10.5061/dryad.dncjsxm2n.

## 3 RESULTS

### 3.1 Comparing sperm longevity between breeding‐coloured and sneaker‐morph males (2012 and 2021)

**FIGURE 1:**
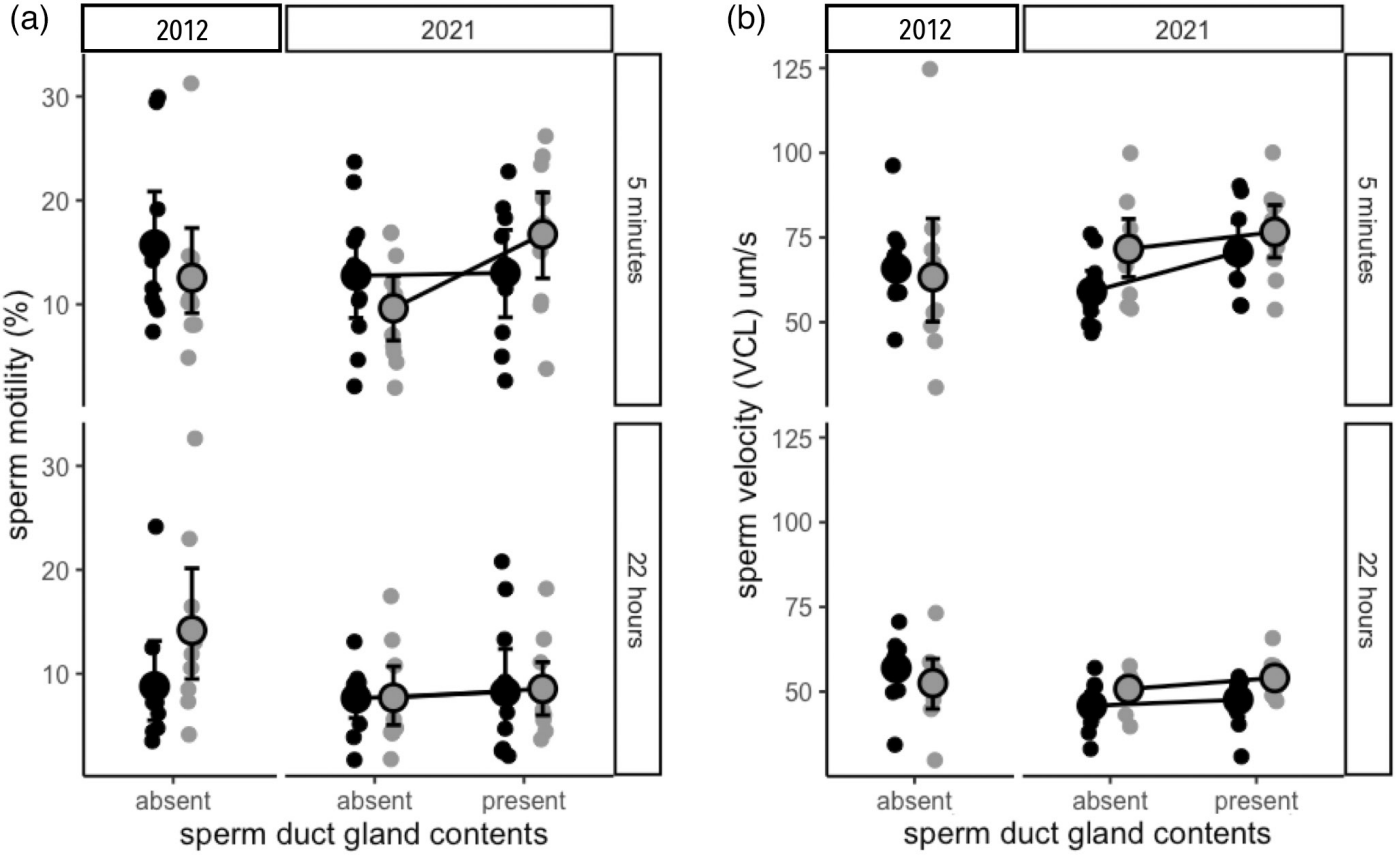
(a) Sperm motility (% motile sperm) and (b) velocity (velocity of the curvilinear path, VCL, μm s^−1^) were measured for sand goby (*Pomatoschistus minutus*) sperm that were activated and kept in 12°C seawater, which was either seawater that contained sperm‐duct gland content (2021) or not (2012 and 2021). Motility was measured approx. 5 min and 22 h after initial sampling. The graphs show nonparametric bootstrapped mean ± CI values (large circles and error bars) and individual data points (small dots) for breeding‐coloured males (black) and sneaker‐morph males (grey). The sperm were tested with sperm‐duct gland contents (mucus) present or absent. Since sneaker‐morph males have no or very small sperm‐duct gland, all sperm‐duct glands came from breeding‐coloured males

Both male morphs had long‐lived sperm. All males had motile sperm at the first measurement, immediately after dissection (Figure 1a). In 2012, nine of the 10 sneaker‐morph males and nine of the 10 breeding‐coloured males still had motile sperm in at least one of the technical replicates after 22 h. Among the three sneaker‐morph males and five breeding‐coloured males that still had sperm suspension left for an extra assay after 48 h, two sneaker‐morph males and two breeding‐coloured males had motile sperm in at least one of the technical replicates collected per male. Hence, measured this way, sperm of the two male morphs did not differ in longevity (chi‐square tests: 5 min: *X* ^2^ = 0, *p* = 1.0; 22 h: *X* ^2^ = 0, *p* = 1.0; 48 h: *X* ^2^ = 0.53, *p* = 0.47). Similarly, based on the 5‐min and 22‐h recordings in 2021, and measured as the estimated time when only 5% of the sperm that were originally motile would still be motile, sperm longevity did not differ between the morphs (*lm*; male morph: *F*_1,18_ = 0.08, *p* = 0.78). Sperm longevity was estimated to be 195,755 s (54.4 h) for sperm from breeding‐coloured males and 203,184 s (56.4 h) for sperm from sneaker‐morph males.

### 3.2 Comparing sperm motility and velocity between breeding‐coloured and sneaker‐morph males (2012)

No significant difference was found in either sperm motility (*lmer*; male morph, *F*_1,35_ = 0.12, *p* = 0.74) or sperm velocity (*lmer*; male morph, *F*_1,35_ = 0.40, *p* = 0.53; Table 1, Figure 1) between breeding‐coloured and sneaker‐morph males. Time (5 min vs. 22 h) had no effect on sperm motility (*lmer*; time, *F*_1,35_ = 1.08, *p* = 0.31); however, sperm velocity did decline with time, although this effect was not significant (*lmer*; time, *F*_1,35_ = 3.32, *p* = 0.077; Table 1, Figure 1). No interactions were found to affect either sperm motility or sperm velocity.

### 3.3 Comparing sperm motility and velocity between breeding‐coloured and sneaker‐morph males with and without sperm‐duct gland contents (2021)

No significant difference was found in sperm motility between breeding‐coloured and sneaker‐morph males (*lmer*; male morph, *F*_1,75_ = 0.02, *p* = 0.88; Table 1, Figure 1). Presence of sperm‐duct gland contents showed a nonsignificant trend of increasing sperm motility for both sneaker‐morph and breeding‐coloured males (*lmer*; sperm‐duct gland treatment, *F*_1,75_ = 2.83, *p* = 0.096; Figure 1). In this experiment, time was found to significantly decrease sperm motility for both male morphs (*lmer*; time, *F*_1,75_ = 14.28, *p* < 0.001; Table 1, Figure 1). No interactions were found to affect sperm motility.

Differences were more pronounced when comparing sperm velocity. Sneaker‐morph males were found to have higher velocity than breeding‐coloured males (*lmer*; male morph, *F*_1,17.9_ = 5.49, *p* = 0.031; Table 1, Figure 1). Furthermore, velocity increased when sperm were tested in sperm‐duct gland contents for both male morphs (*lmer*; sperm‐duct gland treatment, *F*_1,57.1_ = 7.93, *p* = 0.007; Table 1, Figure 1). Sperm velocity also significantly decreased with time (*lmer*; time, *F*_1,57.1_ = 113.3, *p* < 0.001; Table 1, Figure 1). No interactions were found to affect sperm velocity, showing that the effect of time and sperm‐duct gland treatment was similar for the two male morphs.

### 3.4 Comparing gene expression of testes between breeding‐coloured and sneaker‐morph males (2012)

**FIGURE 2:**
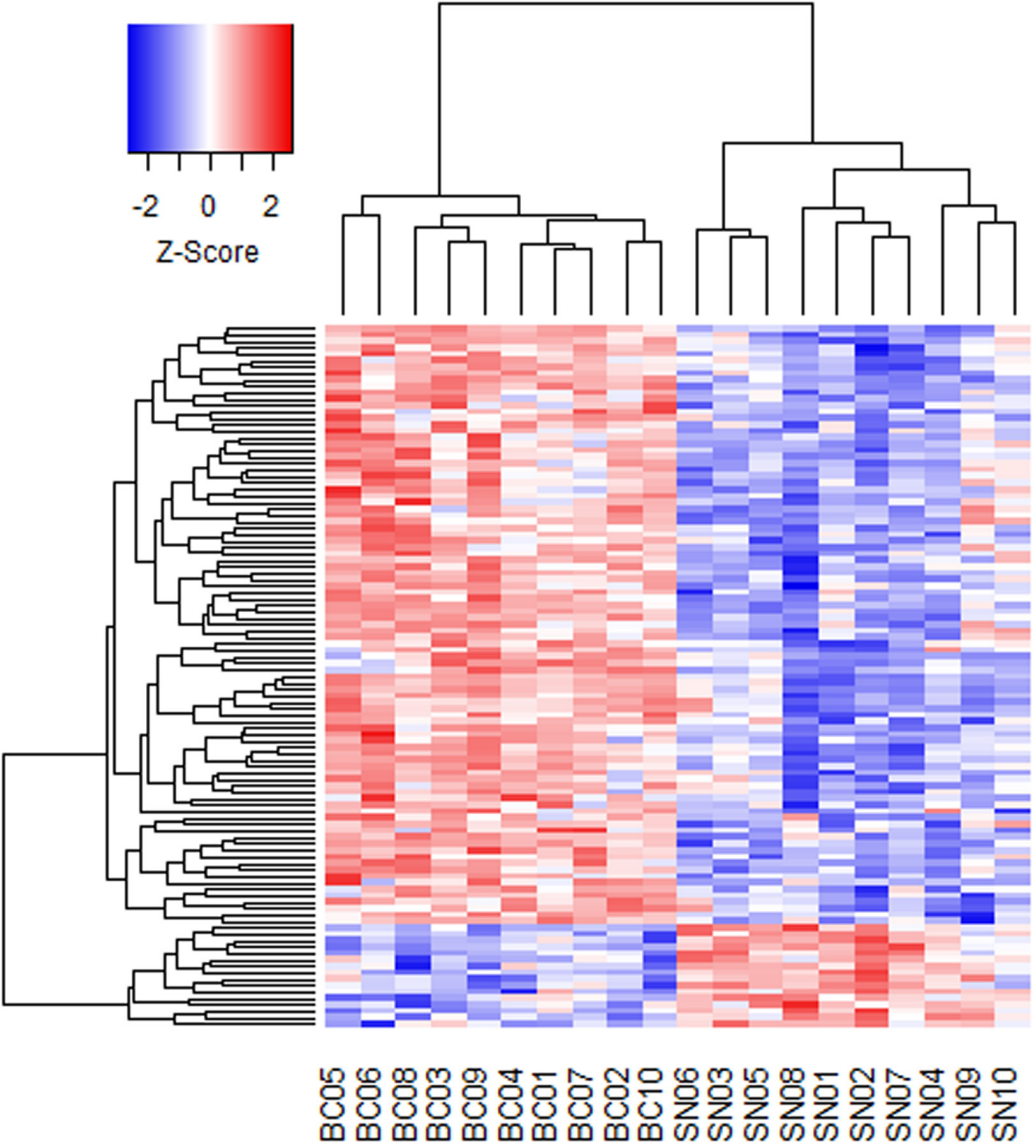
Heatmap of differentially expressed genes between breeding‐coloured (BC) and sneaker‐morph males (SN) of sand goby (*Pomatoschistus minutus*). The expression value of each gene was standardized to the mean (*Z*‐Score). Red colour shows genes that are upregulated, and blue indicates genes that are downregulated within one morph in relation to the other morph. Genes and samples are clustered with complete‐linkage clustering using coolmap from the R package limma (Ritchie et al., 2015)

**FIGURE 3:**
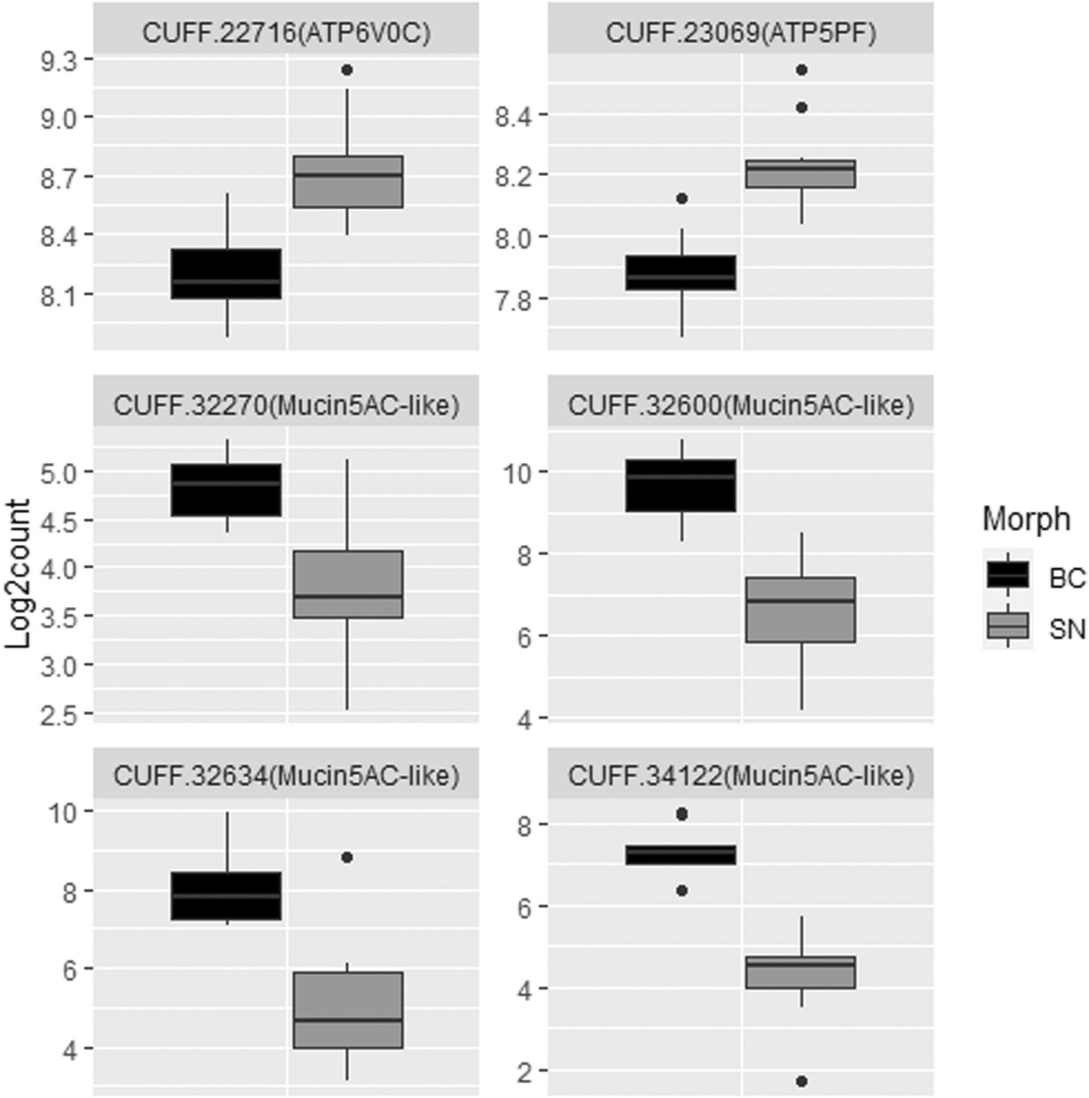
Boxplots of selected differentially expressed genes between breeding‐coloured (BC, in black) and sneaker‐morph males (SN, in grey) of sand goby (*Pomatoschistus minutus*)

We compared gene expression in testes between breeding‐coloured and sneaker‐morph males and found that 109 transcripts differed significantly between the male morphs. Of these, 16 transcripts were upregulated in sneaker‐morph males, and 93 transcripts upregulated in the breeding‐coloured males compared with the other morph males (Figure 2, Table S1,S2). Notably, four of the transcripts that were upregulated in breeding‐coloured males were annotated as mucin genes (mucin5AC‐like; Figure 3).

Functional enrichment analysis of the genes upregulated in breeding‐coloured males identified several categories within the categories Cellular Component and Molecular Function (Table S2), most notably extracellular exosome, and structural molecule activity. For genes upregulated in sneaker‐morph males ‘proton‐transporting ATP synthase activity, rotational mechanism’ was an enriched Molecular Function category and ‘proton‐transporting two‐sector ATPase complex’ was an enriched Cellular Component. However, both of these are just based on two ATP‐related genes (ATP6V0C & ATP5PF; Figure 3) among the 11 genes for which gene names were found.

**FIGURE 4:**
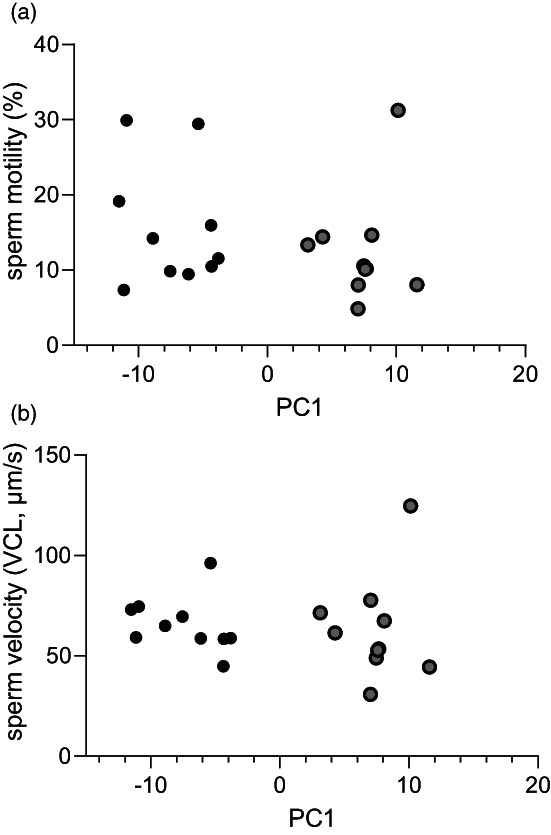
(a) Sperm motility (% motile sperm) and (b) velocity (velocity of the curvilinear path, VCL, μm s^−1^) of sand goby males (*Pomatoschistus minutus*) depicted with the 1st principal component (PC1) of 109 transcripts that were differently expressed between breeding‐coloured (black) and sneaker‐morph males (grey). The sperm performance assays and gene expression were collected in a paired design, from one testis each of the same male. The outlier is sneaker‐morph male SN08. This male is visible in Figure 2 as having a large proportion if its genes downregulated

We found no association between PC1 of gene expression and sperm motility (5‐min measurement) for either male morph, as analysed using PC1 of the 109 differently expressed transcripts as covariate and male morph as factor (ANCOVA; male morph, *F*_1,16_ = 0.10, *p* = 0.76; PC1, *F*_1,16_ < 0.01, *p* = 0.98; interaction, *F*_1,16_ = 0.51, *p* = 0.49; Figure 4a). The same was true for sperm velocity (ANCOVA; male morph, *F*_1,16_ = 0.01, *p* = 0.92; PC1, *F*_1,16_ < 0.01, *p* = 0.99; interaction, *F*_1,16_ = 0.38, *p* = 0.55; Figure 4b).

### 3.5 Comparison of sperm morphometrics between breeding‐coloured and sneaker‐morph males (2021)

We found no difference between breeding‐coloured and sneaker‐morph males in sperm head length (Welch *t*‐test; *t*_17_ = −0.20, *p* = 0.42), flagella length (Welch *t*‐test; *t*_14.76_ = −0.88, *p* = 0.20), sperm total length (Welch *t*‐test; *t*_14.49_ = −0.86, *p* = 0.20) or the flagella length to head length ratio (Welch *t*‐test; *t*_16.86_ = −0.92, *p* = 0.19). Sperm morphometrics did not correlate with sperm velocity (5 min), and male morph had no effect, neither for sperm head length (ANCOVA: sperm head length: *F*_1,15_ = 1.07, *p* = 0.32; male morph: *F*_1,15_ = 1.04, *p* = 0.32; interaction: *F*_1,15_ = 0.21, *p* = 0.66), flagella length (ANCOVA: sperm flagella length: *F*_1,15_ = 0.79, *p* = 0.39; male morph: *F*_1,15_ = 0.81, *p* = 0.38; interaction: *F*_1,15_ = 0.72, *p* = 0.41), total sperm length (ANCOVA: sperm total length: *F*_1,15_ = 0.91, *p* = 0.35; male morph: *F*_1,15_ = 0.80, *p* = 0.38; interaction: *F*_1,15_ = 0.86, *p* = 0.37), or the flagella length to head length ratio (ANCOVA; ratio: *F*_1,15_ = 0.09, *p* = 0.77; male morph: *F*_1,15_ = 0.98, *p* = 0.34; interaction: *F*_1,15_ = 0.07, *p* = 0.80).

## 4 DISCUSSION

We examined whether sperm from males with alternative reproductive tactics, that is breeding‐coloured and sneaker‐morph males, differed in performance and morphology, and whether the tactics are associated with different gene expression in the testes. Sperm competition theory and previous studies suggest that given the differences in fertilisation opportunities of the two breeding tactics, and their differences in breeding behaviour and reproductive morphology, we should expect corresponding differences in sperm performance, sperm morphology and gene expression. However, except for clear differences in gene expression, we found relatively small differences between the two morphs. The sand goby is a fish with remarkably long‐lived sperm, but this trait is similar in breeding‐coloured and sneaker‐morph males. Comparing sperm performance between male morphs, we found that sperm motility did not differ, while sperm velocity was significantly higher for sneaker‐morph males in only one of the 2 years studied. Presence of the sperm‐duct gland contents increased sperm velocity, but equally so for the two morphs. A similar (but nonsignificant) result was found for sperm motility. We found no difference in sperm morphometrics between male morphs and no correlation between these measures and sperm velocity, regardless of male morph. However, more than 100 transcripts were differentially expressed between the morphs, including four mucin genes upregulated in breeding‐coloured males, and two ATP‐related genes upregulated in sneaker‐morph males.

### 4.1 Effects of male morph on sperm motility and velocity

The higher sperm velocity for sneaker‐morph males compared with breeding‐coloured males, which we found in 2021, may reflect selection pressure on sneaker‐morph males to compensate for an unfavourable position or timing of ejaculation, compared with nest‐holding males that have continued access to the nest and whose sperm‐trails are placed in close proximity to any newly laid eggs. Similar results have been found in the cichlid *Telmatochromis vittatus*, in which sneaker males have faster swimming sperm than any of the other three tactics present in this species (pirates, territorials and satellites; Fitzpatrick et al., 2007), in the black goby (*Gobius niger*), in which sperm of sneaker males have higher velocity, viability and ATP content compared with territorial males, and in the dusky frillgoby (*Bathygobius fuscus*), in which sneaker male sperm are longer‐lived and their velocity declines more gradually compared with sperm of nest‐holding males (Nakanishi & Takegaki, 2019). In contrast, the grass goby (*Zosterisessor ophiocephalus*) shows no such differences between male types (Locatello et al., 2007).

Yet, in our study, sperm motility did not differ between male morphs in either year, and sperm velocity was significantly higher for sneaker‐morph males in only 1 year. Social condition can affect sperm traits in fish in as short a time as 4 days (Rudolfsen et al., 2006). We kept males in same‐sex storage tanks for 2–7 days before sampling, and hence, our storage conditions may have contributed to the lack of difference in motility between male morphs. The difference we observed between years in velocity in the different morphs may be a result of unrecorded differences between years in, for example, local population density, size distributions and proportion of sneaker‐morph males. Finally, although we have good reasons for focusing on velocity of the curvilinear path (see Material and Methods), alternative measures of sperm velocity might capture the aspect of sperm movement that is the target of selection in a better way (e.g. Vaz Serrano et al., 2006). Our results highlight that outcomes can differ between very similar studies on the same species. Although this is a well‐understood consequence of sampling error and a justification for repeating experiments (Fisher, 1934), it is particularly relevant in the light of recent explorations of experimental repetition (Clark et al., 2020; Clements et al., 2022).

### 4.2 Effects of sperm‐duct gland contents on sperm motility and velocity

The presence of the sperm‐duct gland contents in the water increased sperm velocity. This was also true for sperm motility, although the result was not significantly different. However, we found no difference between male morphs in the response to the sperm‐duct gland contents, which means that both morphs benefitted equally from having the contents of the sperm‐duct gland mixed into the test water. A similar positive effect of sperm‐duct gland contents on sperm velocity has previously been found in sand gobies (only tested on sperm of breeding‐coloured males: Green & Kvarnemo, 2019), but not in round gobies regardless of male morph (Green et al., 2020). A study on grass goby found that the seminal fluid (including sperm‐duct gland contents) from territorial males had a particularly positive effect on sneaker male sperm (Locatello et al., 2013), while in the black goby seminal fluid improved sperm velocity only for the guarder males' own sperm (Poli et al., 2018). Thus, the specific effects of the sperm‐duct gland contents on sperm motility and velocity appear to vary among species of gobies.

Our experimental design, using sperm‐duct glands from breeding‐coloured males for the sperm assays of both male morphs, reflects the natural spawning situation in which sneaker male sperm would be exposed to the mucus of the breeding‐coloured male. Based on their lack of (or very small) sperm‐duct glands (Kvarnemo et al., 2010; Svensson & Kvarnemo, 2007) and our own observations of sneaker‐morph male behaviour upon entering a nest, it is likely that sneaker‐morph males simply ejaculate straight into the water inside the nest. Whether nest‐holding males do that too, or if they are restricted to only depositing sperm via sperm‐containing mucus trails, is not known. Regardless, mucus production and storage should be a costly adaptation for breeding‐coloured males, which sneaker‐morph males avoid thereby allowing them to form larger testes (Takegaki et al., 2012), while free‐riding on the positive effect of sperm‐duct gland contents provided by the breeding‐coloured nest‐holders.

### 4.3 Effect of male morph on sperm longevity

There was no difference in sperm longevity between the two male morphs, and both morphs showed very long‐lived sperm. This result is in line with previous findings from sand gobies and a few other goby species (Green & Kvarnemo, 2019; Lindström et al., 2021; Nakanishi & Takegaki, 2019). Here, we found that all but two males had motile sperm after 22 h, and some had motile sperm even after 48 h. Comparing sperm movement after 5 min and 22 h, we found no decrease in sperm motility over time and only a slight and nonsignificant decline in velocity in 2012. In 2021, we found significant declines with time in both motility and velocity, but both sperm traits stayed well above zero. Compared with many other fishes, whose sperm stay motile for seconds or minutes (Browne et al., 2015), this is remarkable. It is hard to speculate what selective pressures and steps have allowed such long‐lived sperm to evolve; however, it means that once ejaculated, sperm may retain their fertilisation ability for a very long time as long as they are not swept away by currents or (in the case of sneaker male sperm) removed by vigorous fanning by the nest‐holder (Takegaki et al., 2020). Further, extended sperm longevity is likely to offer a similar function to the sperm‐containing mucus (Marconato et al., 1996), that is, releasing the male from the need to stay close to the spawning female, freeing him to defend the nest from intruders.

### 4.4 Effect of male morph on sperm morphometrics

We found no differences in sperm morphometrics between breeding‐coloured and sneaker‐morph males, for any of the traits that we measured. Also, none of the traits correlated with sperm velocity. This may be explained either by a true lack of difference between male tactics, as found in several other species of fish and other taxa (reviewed in Kustra & Alonzo, 2020), or be a result of our sampling method, that is, sourcing the sperm directly from the testes and not as part of a stripped ejaculate. A study of the plainfin midshipman (*Porichthys notatus*) found that sperm that were sampled from ejaculates were larger in size (head and midpiece) than sperm from the testes (Miller et al., 2019). Nonetheless, based on a multivariate analysis, the morphological traits of plainfin midshipman sperm did not differ between dominant and sneaker males (Miller et al., 2019), a finding similar to our own. In other fish species, differences in sperm shape can be found between male morphs. For example, in the ocellated wrasse (*Symphodus ocellatus*), sneaker males had larger sperm heads, compared with nesting males and satellite males, while tail length did not differ between the three types of males (Alonzo et al., 2021).

### 4.5 Effect of male morph on gene expression

Comparing gene expression in testes, 109 transcripts differed significantly between the male morphs. Of these, 93 transcripts were upregulated in breeding‐coloured males and 16 transcripts were upregulated in sneaker‐morph males, suggesting that there are differences related to testes function between the male morphs.

Despite the few categories that were functionally enriched in each morph, there is some evidence that the differentially expressed genes can allow each morph to reproduce successfully with their given reproductive tactic. Specifically, we found four mucin genes upregulated in breeding‐coloured males, and ATP hydrolysis and catalysis upregulated in sneaker‐morph males. Increased mucin production is likely to aid breeding‐coloured males by providing a good environment for the eggs in their care, and for sperm stored in mucus trails. An increase in genes related to energy use and storage (i.e. ATP) in the sneaker‐morph males may provide their sperm with more energy, matching the 2021 result of a higher sperm velocity for this male morph. Faster swimming black goby sneaker sperm have also been found to contain more ATP than sperm from territorial males (Locatello et al., 2007); however, there was no difference in sperm velocity between male morphs in 2012, the year when gene expression was analysed. Additionally, other differentially expressed genes may contribute to advantages in sperm production or function of each respective morph; however, not enough is known about the process of spermatogenesis in fish and the specific genes involved. For instance, several differentially expressed genes have a known function in spermatogenesis in mammals, for example, KELCH10 (Yan et al., 2004) and Nardilysin (Segretain et al., 2016), but how these genes could contribute to a specific advantage in one morph versus the other cannot be inferred with our current knowledge. When comparing our results to those from the bluehead wrasse (Todd et al., 2018), only two genes were common to the two studies and both were upregulated in territorial/breeding‐coloured males compared with sneaker males: cytochrome P450, family 17, subfamily A, polypeptide 1 (cyp17a1) and steroidogenic acute regulatory protein (star). Both of these genes and also cyp11b1 (our data) and cyp11c1 (Todd et al., 2018) are involved in steroidogenesis. Furthermore, cyp11b1 was also upregulated in testes of territorial midshipman fish, *Porichthys notatus*, compared with sneaker male testes (Arterbery et al., 2010). In combination with these other studies, our results further support that morph‐specific steroidogenic activity in the testes may be a common feature in species with alternative reproductive tactics (Arterbery et al., 2010; Knapp, 1995).

The occurrence of four mucin genes upregulated in the testes of breeding‐coloured males corresponds with the reproductive tactic of the breeding‐coloured males, which build nests, care for eggs, and have large sperm‐duct glands (Kvarnemo et al., 2010; Svensson & Kvarnemo, 2007). However, the result is somewhat surprising since gene expression was analysed in the testes, and not the sperm‐duct glands. Based on histology, it has been assumed that the majority of mucin production is in the sperm‐duct glands (Fishelson, 1991; Scaggiante et al., 1999); however, our results suggest that mucins are also produced, or at least transcribed, in the testes. Further evidence that the testes may produce mucins, which are transported to the accessory glands comes from the enrichment analysis. ‘Extracellular exosome’ and ‘vesicles’ were enriched cell components. Exosomes are vesicles that transport substances from one cell to another distant cell. In humans, mucin5AC has been shown to be transported within exosomes originating in the nasal cavity (Wang et al., 2021); thus, it is possible that mucins are produced in the testes and transported to the accessory glands in exosomes.

### 4.6 Applied importance of sand goby reproductive biology

The sand goby is a valuable model for studies of reproduction in evolutionary ecology (Forsgren, 1999; Svensson & Kvarnemo, 2007) and is one of the noncommercial marine fish for which the genome has been sequenced (Leder et al., 2021). It is also a frequently used model system in applied studies, such as ecotoxicological assays (Asnicar et al., 2018; Kirby et al., 2003; Saaristo et al., 2009), studies of hypoxia (Lissåker & Kvarnemo, 2006; Olsson et al., 2016), eutrophication (Järvenpää & Lindström, 2004) and aquatic noise (Blom, 2017).

Our finding in this paper that breeding‐coloured and sneaker‐morph males have clearly different gene expression profiles provides an important contribution to understanding the underlying genetics of reproductive behaviour, which is essential for evolutionary studies. Furthermore, sand goby sperm are locally adapted and as such form a potential barrier to gene flow (so‐called immigrant reproductive dysfunction), enabling further adaptation (Leder et al., 2021; Svensson et al., 2017). The detailed understanding of sperm function, ecologically as well as evolutionarily, gathered in this paper and in previous research, provides general conceptual tools that may be invaluable in the context of speciation.

Insights gained from basic research can be crucial also in applied research on invasive biology, to better understand and predict species expansion. For example, our work on environmental tolerance of sand goby sperm has been instrumental for answering questions on the very rapid spread over a wide environmental gradient by the highly invasive round goby *N. melanostomus* (e.g. Green et al., 2019; Green, Apostolou, et al., 2021a; Green, Niemax, et al., 2021b). More generally, in the last decades, several species of gobies have become invasive throughout the world, and for most species their reproductive biology is one of the keys to their success (Gertzen et al., 2016; Green, Apostolou, et al., 2021a). One of the newest additions is the Caucasian dwarf goby, belonging to the *Knipowitschia caucasica*‐complex (Borcherding et al., 2021), a species whose evolutionary ecology has not yet been studied. However, given that this species belongs to the sand goby clade, knowledge gained from sand goby may be transferable. The mating systems of the invasive species of gobies are, as in the sand goby, characterized by sperm competition and alternative reproductive tactics with bourgeois males producing a multifunctional mucus (e.g. Green et al., 2020). In the present study, we advance our knowledge of the evolutionary ecology of sperm and mucus of alternative reproductive tactics including related gene expressions.

## 5 CONCLUSIONS

Evolution of alternative reproductive tactics in the light of sperm competition is a timely topic and, as several recent reviews have pointed out, is not yet fully understood (Dougherty et al., 2022; Fitzpatrick, 2020; Kustra & Alonzo, 2020). In the current study, we were particularly interested in differentiating sperm traits between males of alternative reproductive tactics, and access to the sand goby genome allowed us to identify adaptations and gene expression linked to such tactics. Here, we have shown that the breeding system of sand goby results in sperm adaptations that include remarkably high longevity, a positive effect of the mucous sperm‐duct gland content on sperm velocity regardless of male morph, and (in 1 year), the sperm of sneaker‐morph males showed higher velocity compared to that of breeding‐coloured males. Linking these adaptations to gene expression in testes, we find marked differences in expression between male morphs, including steroidogenesis and mucin genes upregulated in breeding‐coloured males, and genes involved in ATP hydrolysis and catalysis upregulated in sneaker‐morph males. Detailed knowledge of models such as the sand goby grows in importance with the increasing anthropogenic stress on the natural world, with much relevance in the applied context. This study develops our understanding of the mechanisms in a marine model system useful for evolutionary studies of reproductive tactics, reproductive isolation, local adaptation and speciation.

## CONFLICT OF INTEREST

The authors declare no conflict of interest.

## BENEFIT SHARING

Benefits from this research accrue from the sharing of our data and results on public databases as described above.

## Supporting information


Tables S1,S2

## Data Availability

We comply with Evolutionary Applications’ policy on data archiving, and data from this study will be made available at the Dryad Data repository.
